# Evidences of Changes in Surface Electrostatic Charge Distribution during Stabilization of HPV16 Virus-Like Particles

**DOI:** 10.1371/journal.pone.0149009

**Published:** 2016-02-17

**Authors:** Juan F. Vega, Ernesto Vicente-Alique, Rafael Núñez-Ramírez, Yang Wang, Javier Martínez-Salazar

**Affiliations:** 1 Biophym, Departamento de Física Macromolecular, Instituto de Estructura de la Materia, IEM-CSIC, Madrid, Spain; 2 Sino Biological, Inc., Beijing, People’s Republic of China; Academia Sinica, TAIWAN

## Abstract

The stabilization of human papillomavirus type 16 virus-like particles has been examined by means of different techniques including dynamic and static light scattering, transmission electron microscopy and electrophoretic mobility. All these techniques provide different and often complementary perspectives about the aggregation process and generation of stabilized virus-like particles after a period of time of 48 hours at a temperature of 298 K. Interestingly, static light scattering results point towards a clear colloidal instability in the initial systems, as suggested by a negative value of the second virial coefficient. This is likely related to small repulsive electrostatic interactions among the particles, and in agreement with relatively small absolute values of the electrophoretic mobility and, hence, of the net surface charges. At this initial stage the small repulsive interactions are not able to compensate binding interactions, which tend to aggregate the particles. As time proceeds, an increase of the size of the particles is accompanied by strong increases, in absolute values, of the electrophoretic mobility and net surface charge, suggesting enhanced repulsive electrostatic interactions and, consequently, a stabilized colloidal system. These results show that electrophoretic mobility is a useful methodology that can be applied to screen the stabilization factors for virus-like particles during vaccine development.

## Introduction

Cervical cancer is found to be associated with human papillomavirus (HPV) infection, with HPV16 being the most common type. The HPV genome encodes six early (E) proteins involved in viral replication and host cell malignant transformation, and two late (L) structural proteins, L1 and L2. Structural studies have shown that the virion, with a diameter of around 50–60 nm, is composed of 360 L1 (~ 55 kDa) protein molecules, assembled as 72 L1 pentamers (capsomeres), and 72 copies of L2 protein located at the central opening of each pentamer [1]. Two types of interaction between the capsomeres have been reported in HPV; either five units can surround a capsomere forming a 5-fold axis (in twelve of the capsomeres), or six units can surround a capsomere forming a 6-fold symmetry (in the sixty others) [2–4]. The L1 C-terminal arms mediate intercapsomeres contacts forming disulfide bonds and loops, which extend from the core to create the contacts and reinserting back into the original pentamer. Heterogeneic eukaryotic expression procedures have been used to produce empty virus-like particles (VLPs), which spontaneously assemble from preparations of recombinant L1 protein into a T = 7 icosahedral lattice with 60 nm in diameter [5]. The L1 VLPs are almost identical both morphologically and antigenically to the native virions [6]. In general, VLPs are useful model systems for the investigation of the structural properties of the viral capsid and, additionally, they are attractive candidates for vaccine development [7]. However, expression of HPV16 L1 proteins often yields irregularly shaped and broadly distributed VLPs with a smaller size (30–50 nm) than the expected (60 nm). Notwithstanding, the HPV16 VLPs can be subjected to reassembly and maturation processes in order to improve structure, thermal properties and immunogenicity. Different techniques have been applied to study these processes, including dynamic light scattering, Western Blot analysis, intrinsic fluorescence, transmission electron microscopy and atomic force microscopy [8–13]. Since the viral protein has a net charge in the solution, it can be assumed that repulsive electrostatic interaction competes with attractive hydrophobic forces until a minimal total free energy is reached when the final size of the VLP is attained. The competition between repulsive and attractive interactions in the kinetic process of formation of virus capsids has been theoretically studied, by applying basic concepts of polymer physics and nucleation-growth theory [14,15]. Other recent theoretical treatments have accounted for the importance of the electrostatic interactions in the assembly of empty viral capsids and found correlations between the electrostatic contributions and the capsid size with experimental determinations of protein-protein contacts [16]. Then, any change in the size of the particles given by an aggregation and redistribution of the protein subunits should be accompanied by a change in the charge distribution and the electrophoretic mobility. Johnson et al. argued in 1973 that the electrophoretic mobility is controlled solely by the surface charge density [17]. Since then, the effects of ionic strength, pH and surfactants in electrophoretic mobility of a number of viruses and VLPs have been studied [18–26], but as far as we know this kind of measurements have not yet been performed to follow the maturation and/or stabilization process.

To evaluate whether certain properties of HPV16 VLPs change during the maturation process, we studied the effect of kinetics by using different experimental techniques. In this work, we used dynamic (DLS) and static light scattering (SLS), transmission electron microscopy (TEM) and electrophoretic mobility (EM). We first validated the use of DLS for measuring the diffusion coefficients and apparent dimensions of VLPs, the latter of which was found to be close to those measured by TEM. We additionally measured the second virial coefficient and the apparent molecular weight by SLS. Finally we obtained the surface electrostatic properties as determined by EM. Using these techniques, we found that HPV16 VLPs remain stable during a period of 1–2 hours at 298 K for the selected ionic strength and pH. After the initial induction time, the size, the second virial coefficient, the molecular weight and the electrophoretic mobility, change dramatically with time, suggesting severe alterations in net surface charges and/or their distribution.

## Materials and Methods

### Production and purification of HPV16 virus-like particles

The HPV16 VLPs were kindly provided by Sino Biological, Inc. (Beijing, China), and produced with the system of insect cells infected with HPV L1 expressing baculovirus. Basically, HPV16 L1 gene was inserted into a baculovirus expression vector and transfected into insect cells expressing the HPV L1 proteins which self assemble into VLPs. After multi-step purification process based on chromatography and filtration, the purified HPV16 VLP products can reach at least 95% purity, and stored in aqueous histidine buffered solution at– 80°C until use. The production and purification protocols are essentially those described elsewhere [27–29]. The cryogenic vials were transferred from the– 80°C freezer into a pre-equilibrated cool box with ice to facilitate transport and manipulation. Subsequently the vials were allowed to heat to room temperature (T = 298 K), and manually agitated to enable the thawing process. Right before the whole liquid is completely thawed the vials were placed into the cool box, being ready to further manipulation. The reconstituted purified HPV16 VLPs in solutions containing 0.5 M NaCl, 50 mM sodium citrate, and 1 mM CaCl_2_ (pH 6) were used for all experiments. The initial protein concentration of HPV VLPs in bulk solution was determined by UV absorbance (c = 0.4 mg⋅mL^-1^). Water for all buffers and dilutions was obtained from Milli-Q water purification system (EMD Millipore, Billerica, USA).

### Dynamic light scattering

DLS electric field correlations have been obtained for the initial, intermediate and final states of the VLP treatment using the Zetasizer Nano ZS (Malvern Instruments, Worcestershire, UK) at T = 298 K during a period of 48 hours, and with a 12 μL quartz batch cuvette (Malvern Instruments ZEN2112). Internal control of the experiments was performed with aqueous solutions of standard polystyrene beads (Thermo Scientific Inc., Waltham, MA, USA and Malvern Instruments, Worcestershire, UK). The Nano ZS instrument incorporates non invasive backscattering (NIBS) optics and homodyne detection, to avoid artifacts in the determination of the size of the particles. This technique measures the time-dependent fluctuations in the intensity of scattered light that occur because particles in solution undergo Brownian motion. The intensity fluctuations measured are used to produce the scattered intensity time correlation function, *g*_2_(*τ*). The time-dependence autocorrelation function of the photocurrent was acquired in the solutions at variable concentration (c < 0.4 mg⋅mL^-1^) and for fixed ionic strength (0.5 M NaCl), every 10 s, with 15 acquisitions for each run. The sample solution was illuminated by a λ_0_ = 633 nm laser at a constant power, and the intensity of light scattered at an angle of θ = 173° was measured by an avalanche photodiode. The Siegert equation expresses the relation between the normalized time correlation function of the scattered intensity *g*_2_(*τ*) and the normalized time correlation function of the electric field *g*_1_(*τ*) [30]:

$${\text{g}}_{2}(\tau )=\text{B}+{\beta [\text{g}}_{1}{\left(\tau )\right]}^{2}$$ (1)

where *τ* is the lag time, *B* is the baseline and *β* (≤1) is a coherence factor that accounts for deviations from the ideal correlation and the experimental geometry.

In the more realistic case, for polydisperse samples, a broad distribution of diffusion coefficient takes place, and then *g*_1_*(τ*) is given by:

$${\text{g}}_{1}\left(\tau \right)=\int_{0}^{\infty }\text{G}\left(\Gamma \right){\text{e}}^{\left(-\Gamma \tau \right)}\text{d}\Gamma$$ (2)

where G(Γ) represents the functional form of the distribution of decay rates Γ. The determination of G(Γ) is not a trivial problem. The cumulant analysis allows one to determine the first few moments of the distribution. We have applied the cumulant analysis by a non-linear fitting procedure using the program SEDFIT (from http://www.AnalyticalUltracentrifugation.com), in order to overcome the shortcomings of the traditional linear fitting for podydisperse samples [31,32]. The corresponding cumulant for the field autocorrelation function can be written as:

$${\text{g}}_{\text{1}}\left(\text{t}\right)\;=\;{\text{e}}^{(-\Gamma \text{τ})\;}\left[\text{1+}\frac{{\text{μ}}_{2}{\text{τ}}^{\text{2}}}{2}\right]\;$$ (3)

where Γ = D_z_⋅q^2^ is the average decay rate with D_z_ being the z-average diffusion coefficient and q = (4πn/λ_0_)sin(θ/2) corresponding to the magnitude of the scattering vector, n the refractive index of the solution, λ_0_ the wavelength of the laser, and θ the scattering angle. In Eq (3), μ_2_ is the variance of the distribution, and the polydispersity index is defined as Q = μ_2_⋅Γ^-2^. Once the decay rate Γ is obtained, it is used to obtain D_z_ and the size distribution by taking advantage of the Stokes-Einstein relationship that relates Γ and, alternatively, the diffusion coefficient to the hydrodynamic diameter, d, of the object that undergoes translational motion as:

$$\text{d}=\frac{{\text{k}}_{\text{B}}\text{T}}{3\pi \eta \text{D}}$$ (4)

In Eq (4), k_B_ is the Boltzmann’s constant, T is the absolute temperature (T = 298 K), and η is the solvent viscosity, η = 0.935 cP. This equation considers the particles as rigid spheres with a diameter related to the translational diffusion coefficient, which, in this instance, depends on the size and conformation of the particle at a given temperature and solvent viscosity. All this process allows one to obtain the average z-size. The weight and number size averages, d_w_ and d_n_, respectively, are used for the interpretation of the results [33].

### Static light scattering

SLS data were obtained using the Zetasizer Nano ZS apparatus. The measurements for different sample concentrations in the dilute regime for solutions were obtained at T = 298 K. The results were fitted by linear regression to the Zimm equation [34]:

$$\frac{\text{Kc}}{{\text{R}}_{\theta }}=\left[\frac{1}{{\text{M}}_{\text{w}}}+2{\text{B}}_{22}\text{c}\right]\frac{1}{\text{P}\left(\theta \right)}$$ (5)

where M_w_ is the weight average molecular weight of the solute, B_22_ the second virial coefficient, and c its concentration in g⋅mL^-1^. K is the optical constant given by:

$$\text{K}=\frac{4\pi^{2}{\text{n}}_{0}^{2}}{{\text{N}}_{\text{a}}\lambda_{0}^{4}}{\left(\frac{\text{dn}}{\text{dc}}\right)}^{2}$$ (6)

where N_a_ is the Avogadro number, and dn/dc is the specific refractive index increment of the solution, which was set at 0.185 mg⋅mL^-1^, the corresponding value for proteins, and the laser wavelength, λ_0_ = 633 nm. The Rayleigh ratio, *R*_θ_, was calculated by subtracting the solvent intensity from the solution intensity (I_A_ = I_Solution_-I_Solvent_) using toluene as the standard (I_T_) for which R_T_ = 1.41×10^−5^ cm^-1^ at T = 298 K and λ_0_ = 633 nm [35]:

$${\text{R}}_{\theta }=\frac{{\text{I}}_{\text{A}}{\text{n}}_{\text{0}}^{\text{2}}}{{\text{I}}_{\text{T}}{\text{n}}_{\text{T}}^{\text{2}}}{\text{R}}_{\text{T}}$$ (7)

where n_0_ and n_T_ are the refractive index of the solvent and toluene, respectively. Each final *R*_θ_ data point was based on averaging not less than 10 statistically consistent measurements. Finally, P(θ) in Eq (5) is the shape factor, which embodies the angular dependence of the sample scattering intensity that occurs when the particles are big enough to accommodate multiple photon scattering. When the particles in solution are much smaller than the wavelength of the incident light, multiple photon scattering is avoided. Under these conditions, the angular dependence of the scattering intensity is lost and P(θ) takes the value of 1 (in our case, as a first approximation, for spherical shells of expected radius of gyration, R_g_, of around 15–25 nm, it takes a value of 0.90–0.95). The use of this simple approximation assumes that the third and higher virial terms contribute only negligibly to the measurements; this assumption can be considered valid under the low protein concentrations used in our experiments. Additionally to the SLS measurements, DLS experiments have been performed at each concentration, in order to test the stability of the samples during the SLS experiments.

### Transmission electron microscopy

HPV16 VLPs from solutions at different dilutions were adsorbed directly onto glow-discharged 400 mesh carbon-coated grids. Following negative staining with 2% (w/v) uranyl acetate, the samples were visualized in a JEOL JEM-2100 electron microscope (JEOL Ltd., Tokyo, Japan) operating at 200 kV. The size of the particles was obtained by taking the geometric mean of two orthogonal measurements. To obtain the number size distribution histograms, a minimum of 100 particles per sample was measured, using a binning interval of 2 nm. Fractional frequency was calculated by dividing the particle count within a size interval by the total particle count from the sample.

### Electrophoretic mobility measurements

EM was measured in the Zetasizer Nano ZS apparatus, which uses phase analysis light scattering (PALS). In this application of the technique, a voltage is applied across a pair of electrodes placed at both ends of a universal disposable capillary cell containing the particle dispersion. Disposable polycarbonate folded capillary cells with gold plated beryllium-copper electrodes (Malvern Instruments DTS1060) were used to perform the measurements. Charged particles are attracted to the oppositely charged electrode, and their velocity was measured and expressed per unit field strength as the EM, μ_e_. A standard polystyrene latex particles in aqueous buffer (pH 9) with an assigned z-potential value of (– 42.0 ± 4.2) mV (Malvern Instruments DTS1235) was used to verify the performance of the instrument. Measurements were carried out in aliquots of the VLPs stock solution. The measurements were performed both as a function of time in continuous mode at T = 298 K, and also in batch mode at t = 0, 14 and 48 hours, in samples of c = 0.1 mg⋅mL^-1^. PALS is an especially interesting technique to be applied in the case studied here, as it permits to accurately measure samples with low particle mobility in solutions with high conductivity (53.5 ± 0.5 mS⋅cm^-1^). Due to the high conductivity of the solutions the monomodal analysis has been applied for the long continuous mode experiment. This type of analysis does not produce EM distributions but results in a faster experiment, avoiding sample and electrode degradation. Additionally, reduced voltages have been used (in the range 8–10 V). In some case the general purpose analysis mode has also been used in order to test the quality and reproducibility of the experiments.

## Results and Discussion

**Fig 1:**
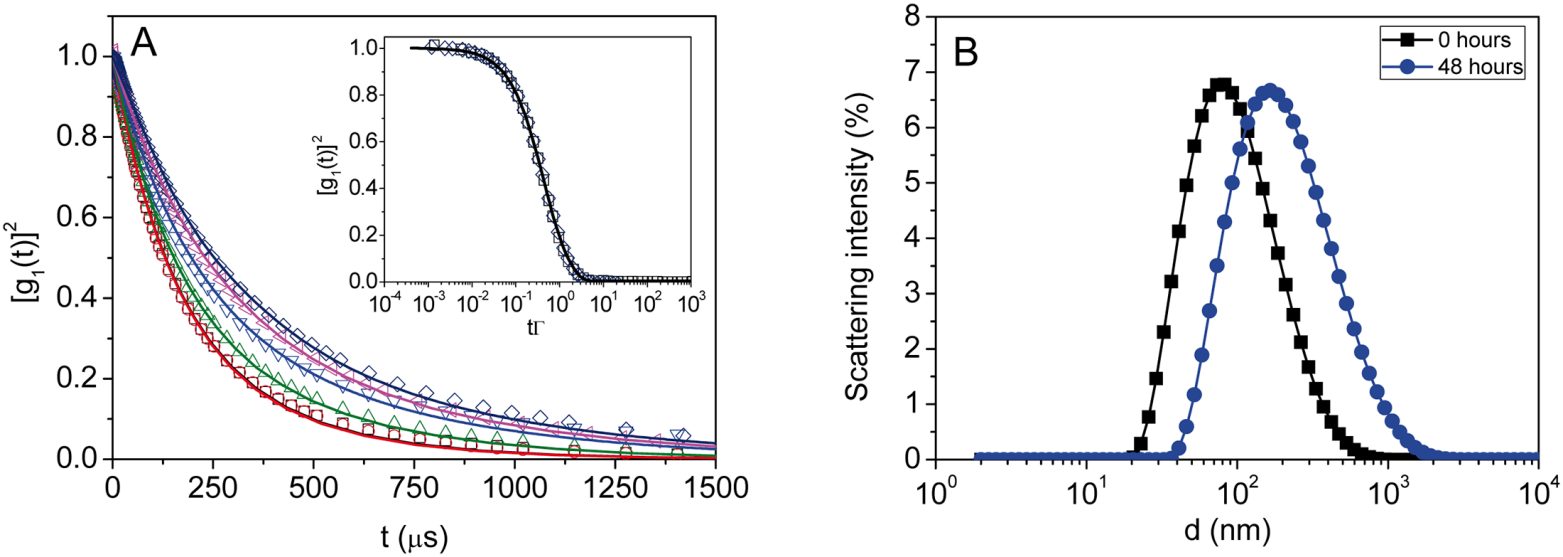
Experimental results obtained from Dynamic Light Scattering experiments. (A) Squared electric field time autocorrelation function, [g_1_(t)]^2^, of the HPV16 VLPs at T = 298 K versus time: (◻, black) 0 h, (○, red) 1 h, (△, green) 2 h, (▽, blue) 6 h, (◁, magenta) 12 h, (▷, cyan) 24 h and (◇,navy) 48 h. The lines represent the cumulant fits given by Eq (3) of the experimental results. The insert shows the fit for initial (0 h, ◻) and final (48 h, ◇) samples in the log-time scale reduced coordinates (tΓ). (B) Intensity distributions determined from DLS results for (∎) initial and (●, blue) final VLPs. Each measurement is an average of 15 scans.

DLS results are presented as the squared electric field autocorrelation function, [g_1_(t)]^2^, for samples treated at different times. Fig 1A shows the values obtained for [g_1_(t)]^2^ for the VLPs studied during the whole period of time (48 hours) at T = 298 K for a sample of c = 0.4 mg⋅mL^-1^. A clear delay of [g_1_(t)]^2^ is observed as time increases. Due to the polydispersity of the samples special care should be taken with the data treatment to determine the exact particle size. The cumulant analysis given by Eq (3) has been shown to be suitable for obtaining the mean sizes of the VLPs from the DLS autocorrelation functions, and the results are listed in Table 1 (see S1 Fig). The fit of the experimental results to Eq (3) is outstanding, since the values of the coefficient of determination, R^2^, are higher than 0.99 in all cases, giving rise to a standard error of the parameters lower than 1%.

**Table 1:** Results of the fit to Eq (3) to DLS data of the HPV16 virus like particles studied.

Sample	I (kcps)	B (s.e.<0.1%)	β (s.e.<0.5%)	Γ⋅10^3^ (1⋅μs^-1^) (s.e.<0.1%)	μ_2_⋅10^6^ (1⋅μs^-2^) (s.e.<1%)	R^2^
0 h	1606	1.0004	0.915	2.69	2.4	0.9998
1 h	1563	1.0003	0.916	2.68	2.6	0.9997
2 h	1471	1.0403	0.865	2.44	2.2	0.9976
6 h	917.3	1.0293	0.856	1.90	1.8	0.9981
12 h	529.7	1.0293	0.790	1.70	1.4	0.9980
24 h	199.7	1.0701	0.789	1.60	1.1	0.9925
48 h	107.5	1.0110	0.880	1.45	0.68	0.9997

s.e. is the standard error of the fit.

**Fig 2:**
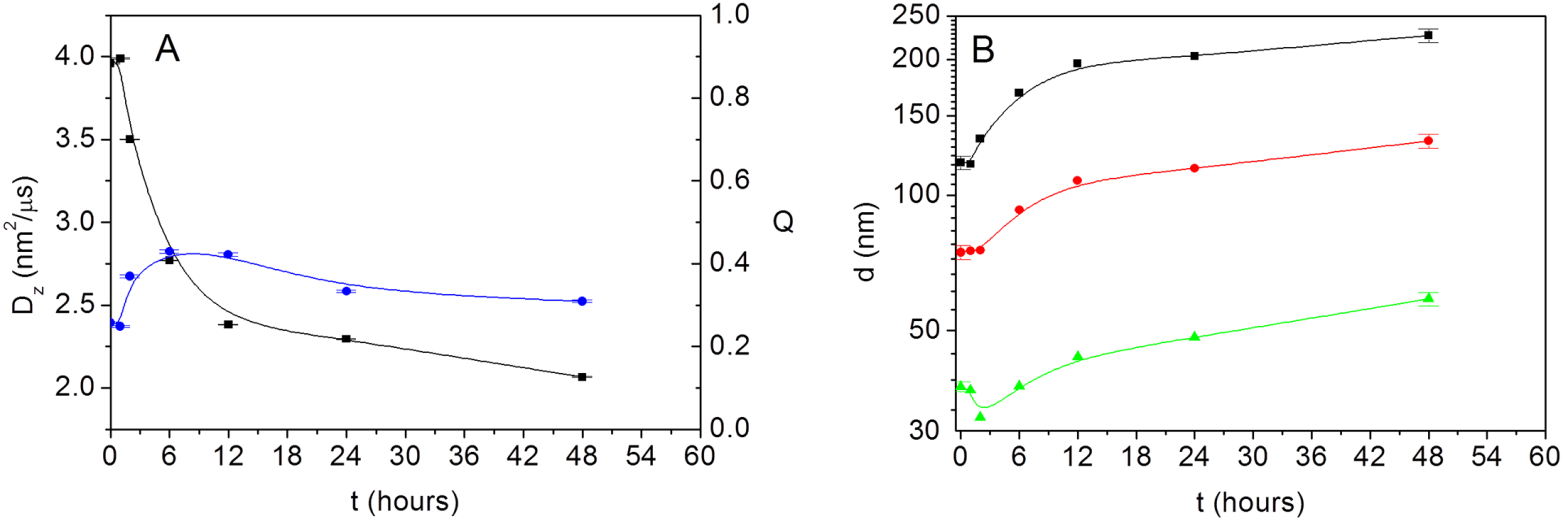
Hydrodynamic properties of virus-like particles. (A) Time evolution of (∎, black) z-average diffusion coefficient (D_z_ = Γ⋅q^-2^) and (●, blue) polydispersity (Q = μ_2_⋅Γ^-2^) obtained from the cumulant analysis (the error bars indicate the standard error of the fit). (B) Average sizes d_z_ (∎, black), d_w_ (●, red) and d_n_ (▲, green) obtained from the cumulant analysis and Eq (8) (the error bars indicate the standard deviation of three independent measurements for initial and final samples).

As it can be observed in Fig 2A, after an induction time of around 1–2 hours, during which hydrodynamic properties remain constant, a decrease of the decay rate, Γ, with time is obtained, which directly leads to a decrease in the diffusion coefficient, D_z_, with time. Also, a slight increase in polydispersity index, Q, is seen just for the initial stages of the process. However, as time proceeds Q values seem to stabilize up to the similar values as those obtained for the initial sample (around a moderate value of Q = 0.32–0.33). Additionally, the goodness of the fits indicates unimodal distributions of diffusion coefficients in all of the cases. In fact, it is also possible the application of a more complex procedure in order to obtain the complete particle size distribution from the autocorrelation function. Such analysis requires the application of inverse Laplace transform by Thikonov analysis [36]. For that, we have also used the program SEDFIT, and the results are shown in Fig 1B for the initial (0h) and the final (48 h) samples. These results demonstrate that the VLPs are well dispersed in the conditions studied here and that no traces of capsomeres or aggregates there exist in the sample.

The main features affecting the Brownian diffusion of colloidal suspended particles are their sizes and the balance between hydrodynamic and electrostatic forces among them. Then, the changes observed in [g_1_(t)]^2^ as the function of time should be attributed to an aggregation mechanism leading to an increase of the size of the VLPs. The average diffusion coefficient obtained from the cumulant analysis is related to the so-called z-average diameter, d_z_, upon application of Eq (4), which is an intensity-averaged size. However, the corresponding weight and number averages of the distribution, d_w_ and d_n_, respectively, are more physically sound, as they can be compared with the values obtained by means of alternative experimental techniques (for example, size exclusion chromatography and transmission electron microscopy). This comparison requires the assumption of a shape of the distribution. The simpler case is the log-normal distribution, which is most likely fine as it can be observed in Fig 1B. In this case there exists a simple relationship among z-, weight- and number-averages by means of the polydispersity factor, Q. Assuming this specific shape distribution, we have made use of the relationships obtained by Thomas [33] given by the expressions:

$${\text{d}}_{\text{w}}=\frac{{\text{d}}_{\text{z}}}{{\left(1+\text{Q}\right)}^{2}};{\text{d}}_{\text{n}}=\frac{{\text{d}}_{\text{z}}}{{\left(1+\text{Q}\right)}^{5}}\;$$ (8)

The results obtained for d_z_, d_w_ and d_n_ have been plotted against time in Fig 2B. It can be clearly observed that the process gives rise to a 2-fold increase of the size of the VLPs. Additional DLS measurements of the initial (between 0–2 hours) and final (48 hours) states have been performed for different aliquots within the concentration range c = 0.01–0.4 mg⋅mL^-1^. The values of the diffusion coefficients, and then of the hydrodynamic sizes are highly reproducible. The autocorrelation coefficients obtained can be found in S2 and S3 Figs. The reproducibility of the experiments is quite satisfactory, with standard deviations, σ, around 3–5%. In numbers the size (d_n_) of the particles increases from ∼ 29 ± 1 nm to around ∼ 58 ± 2 nm (see S1 Table for additional information). It is worthwhile to mention that during the process a decrease of the total integrated intensity values is observed for the selected sample concentration and the laser power used (see Table 1). As both are kept at the same corresponding values, the intensity variation also gives us a direct probe of the kinetics of the process. The total integrated intensity depends not only on the actual size of the particles, but also on the balance between hydrodynamic and electrostatic forces in the solution. This result may hint the presence of some interesting changes in the thermodynamic state of the VLP colloidal solution.

**Fig 3:**
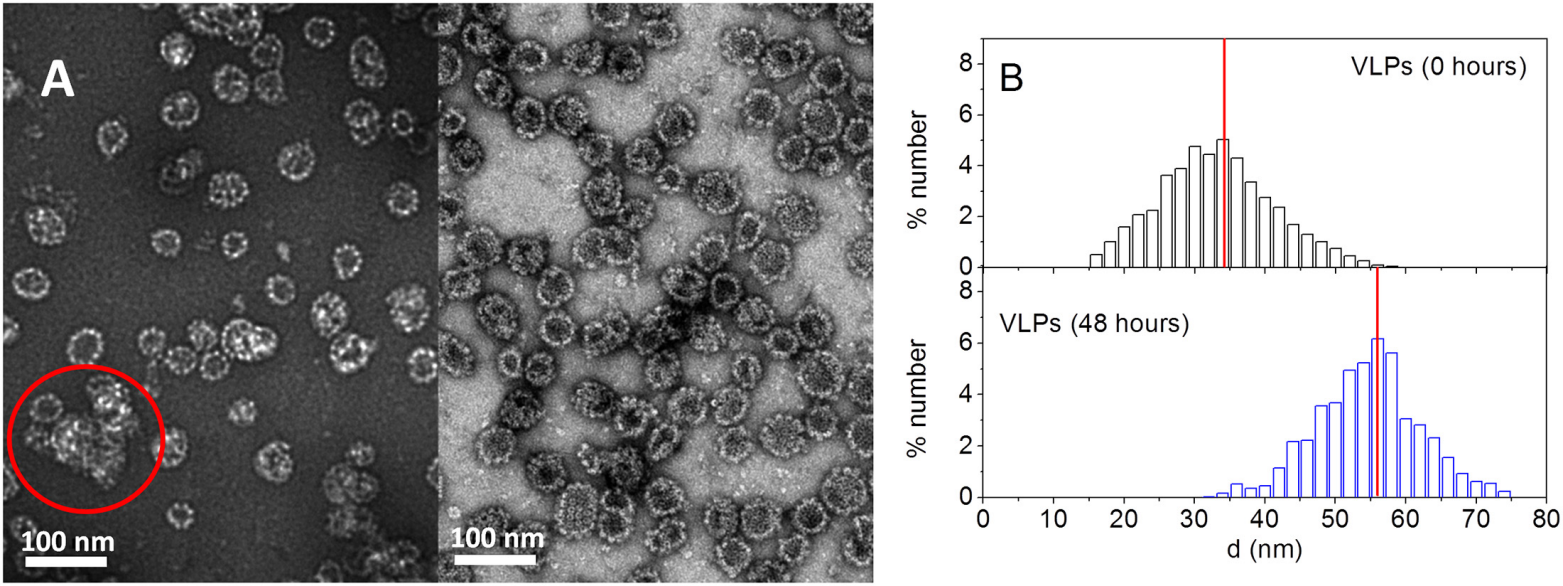
Virus-like particles before and after the stabilization process. (A) Transmission electron micrographs of HPV16 VLPs in the initial (left) and final states (right). (B) Number averaged size histogram obtained from the pictures. The vertical lines correspond to the number averaged results obtained from the DLS analysis and Eq (8).

TEM imaging of the VLPs can provide us with information about the state of the VLP samples before and after the aggregation process. In Fig 3A representative images of the initial and final VLPs obtained by this technique are observed. The dark centers in the empty capsids arise from infiltration of the staining agent into the particles. Initial VLPs (left picture in Fig 3A) appear to be significantly smaller than final ones (right picture in Fig 3A). Furthermore, a substantial portion of initial particles exists as clusters, as it can be also clearly seen in the left micrograph of Fig 3A. The aggregation of VLPs during the preparation of samples for TEM analysis has been also recently reported by other authors [10]. Size histograms (in number) from these pictures can be obtained. If the distribution is monomodal, it can be assumed that particles with one general morphology are present. However, their diameters vary around a mean value, most likely due to a different degree of maturation [37]. The results of the analysis of the TEM images are given in Fig 3B. The histograms confirm the results obtained by DLS. Indeed an increase from around an average of 32 nm to 56 nm is observed (they compare well with ∼ 29 ± 1 nm to ∼ 58 ± 2 nm obtained by DLS). These results coincide with those obtained for the initial and final states by DLS when the averages are expressed in number of particle sizes (see Fig 2B). It should be also noted the similarities between the final VLPs and the expected size of the virus of around 60 nm [1].

**Fig 4:**
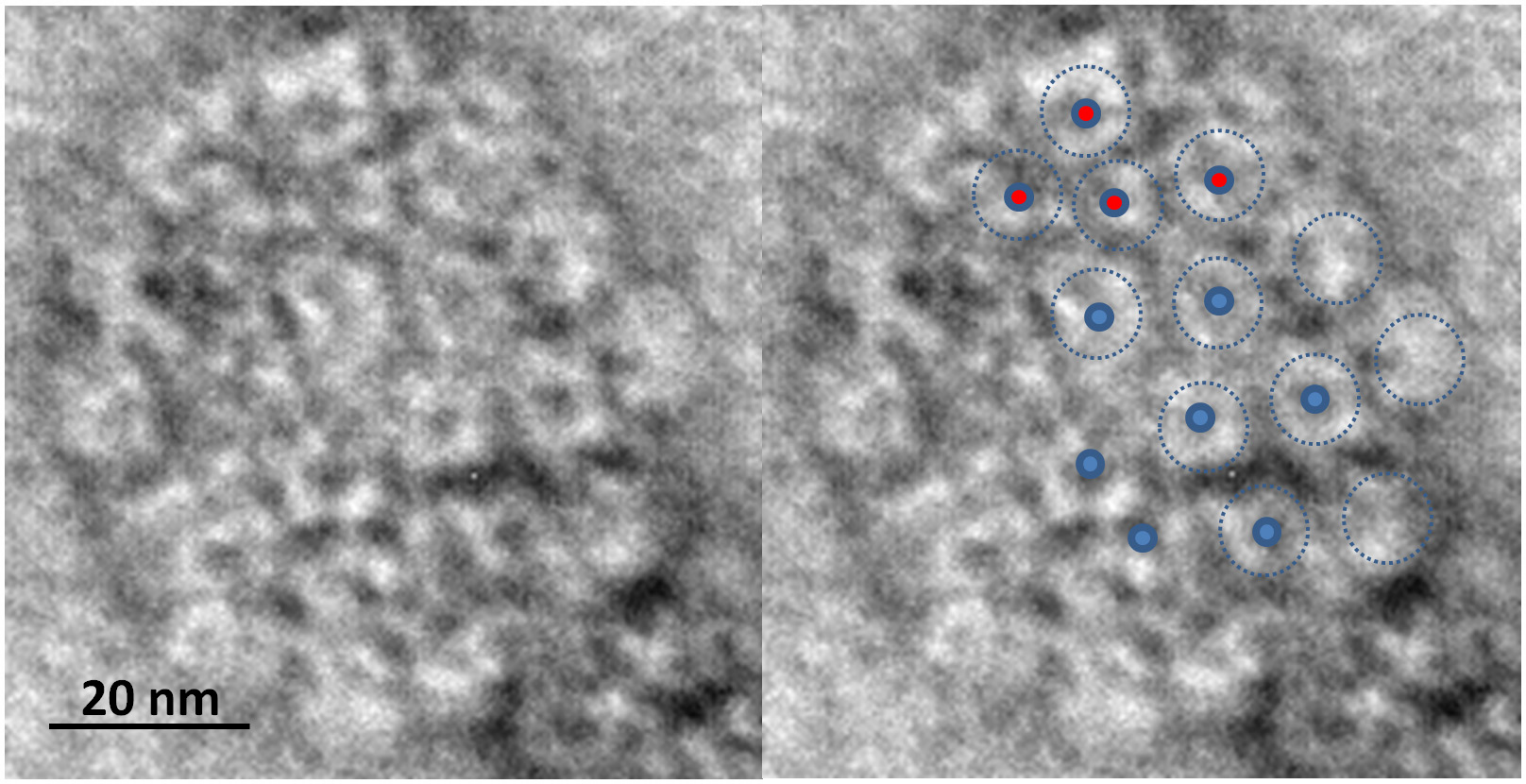
Capsomeres’ 5- fold and 6-fold symmetry of a virus-like particle. A HPV16 VLP at the final state of the stabilization process (48 hours). The dots and dotted circles on the image at the right indicate the positions of the different groups of capsomeres with either a 5-fold or 6-fold symmetry. The size of the particle is ∼ 60 nm.

Although the structure of the mature VLPs studied here is still far from perfect, it is possible to detect some particles with the expected 5- and 6-fold symmetries of the capsomeres, as it can be observed in the micrographs of Fig 4. These interactions are possible due to the flexible C-terminus of the L1 protein subunits, which extends into the nearest capsomeres where they are anchored by a disulfide bond [3].

**Fig 5:**
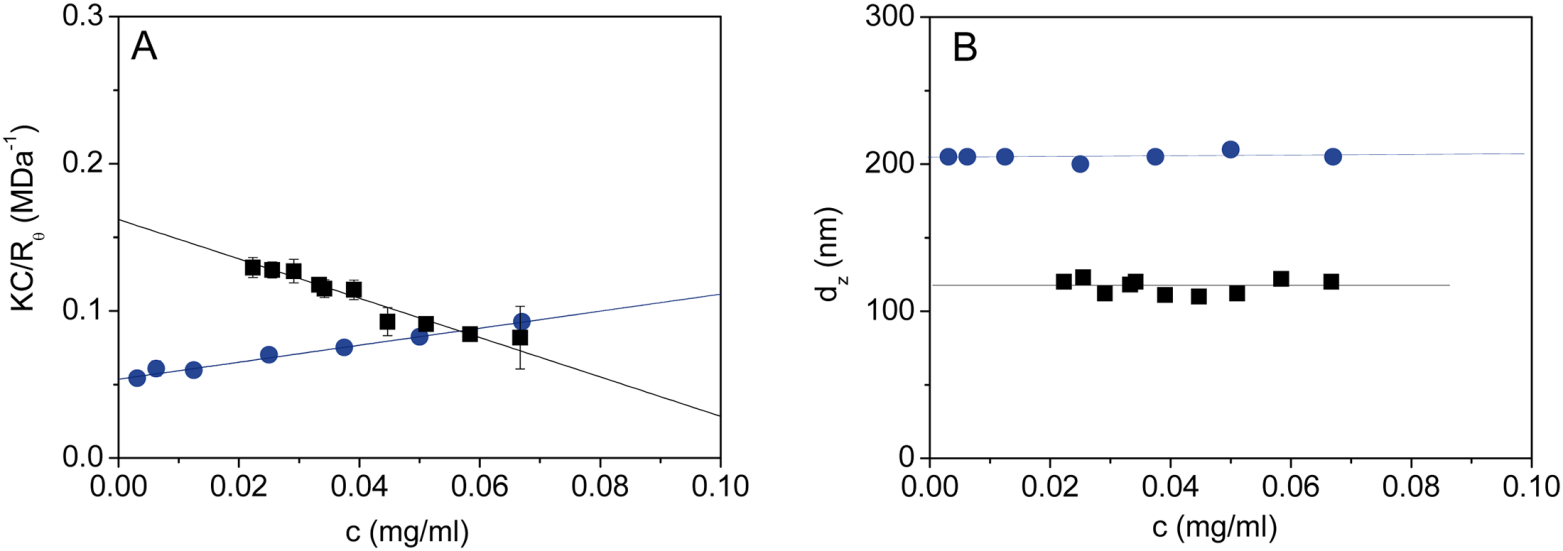
Experimental results obtained from Static Light Scattering experiments. (A) Debye plot of HPV16 VLPs from SLS measurements at T = 298 K for determination of the VLPs molecular weight and the second virial coefficient: (∎, black) 0 hours (R^2^ = 0.9735) and (●, blue) 48 hours (R^2^ = 0.9806). The error bars indicate the standard deviation obtained from at least 15 scans. (B) Z-average size, d_z_, obtained from DLS cumulant analysis during SLS measurements for each concentration. The standard deviation obtained for d_z_ is less than 3%.

As judged by the results obtained from DLS, the VLPs remain stable around the first 1–2 hours at T = 298 K. A similar lag phase has been also reported for the kinetics capsid formation from L1 protein in the case of HPV11 VLPs [38]. After this period of time an aggregation (morphological or structural rearrangement) mechanism takes place, giving rise to an increment in the size of the VLPs. This fact, together with the unimodal nature of the size distributions of the initial and final states and the moderate values of size polydispersity (Q ∼ 0.3) has lead us to perform measurements of the light scattering intensity in static mode in order to detect differences in particle interactions and/or molecular weights between the initial and final VLPs. In Fig 5A the Debye plot of both initial and final samples is observed, and clear differences are noticeable. First of all, for higher concentrations (c > 0.06 mg⋅mL^-1^ as used for DLS experiments) the value of K⋅c/R_θ_ is lower in the initial sample than in its final state. This means that the intensity collected for a given concentration decreases with time, in agreement with the DLS experiments above (see the values of collected intensity, I, in Table 1). Moreover, the intercept of the straight lines is the reciprocal value of the weight average molecular weight, 1/M_w_, following Eq (5). From the values of the intercept, values of 6.2 ± 0.1 MDa and 18.4 ± 0.4 MDa are obtained for the initial and final states, respectively, accounting for a 3-fold increase in the molecular weight of the VLPs at the end of the process. It should be noted that the final determined average molecular weight nicely approaches to the theoretical value of 19.8 MDa corresponding to T = 7 capsids with 360 L1 protein units (each at 55 kDa) and 72 pentamers (each at 275 kDa) [1]. It should be noted that DLS can only provide approximate molecular weight for empty capsid, as it is indicated by the limitation of the technique in differentiating filled global protein versus well-packed VLP with internal space empty. Here we recall that DLS experiments have been performed in parallel with SLS measurements, as explained in the experimental section. In Fig 5B it can be clearly seen that no changes in the diffusion coefficient or the hydrodynamic size occur during the experiments within the range of concentration explored (see the autocorrelation function results obtained for each concentration in S3 Fig). The results in Fig 5B show that the diffusion coefficient is larger in the later, as it was seen previously. Additionally, the cumulant analysis demonstrates that the size of the final samples is closely twice the value of that shown by the initial VLP samples, as it was demonstrated in the previous kinetics experiments in Fig 2.

Finally, the slope of the straight lines in Fig 5A (corresponding to the fit to Eq (5)), gives rise to an estimation of the second virial coefficient, B_22_, which is an empirical parameter that accounts for the ideality of bio-macromolecular dilute solutions. A quantitative understanding of B_22_ is given by the thermodynamic equilibrium solution theory in which this quantity is related to the potential of mean force, W(r), which describes all of the interacting forces between two protein molecules in infinitely dilute solution [39], including the *repulsive* electrostatic interaction, and the *attractive* van der Waals interaction in combination with hydrophobic and excluded volume or steric forces [40–44]. These interactions can be measured and characterized in different ways. The potential between particles of radius r_0_ at the equilibrium is related to thermodynamics by means of B_22_, which characterizes the pair wise interactions, and defined as:

$${\text{B}}_{22}=-\frac{2\pi }{{\text{M}}_{\text{w}}^{2}{\text{N}}_{\text{a}}}\int_{{\text{r}}_{0}}^{\infty }\left[{\text{e}}^{-\text{W}\left(\text{r}\right)/{\text{k}}_{\text{B}}\text{T}}-1\right]{\text{r}}^{2}\text{dr}$$ (9)

Qualitatively, positive values of B_22_ indicate a mutual repulsive interaction between the particles, while negative values an attractive interaction. As it can be observed in Fig 5A, B_22_ is negative for the initial sample (B_22_ = − 0.0023 ± 0.0001 mol⋅mL⋅g^-2^) and positive in the final state (B_22_ = 0.00057 ± 0.00003 mol⋅mL⋅g^-2^). What this means is that at the initial state attractive forces do exist among the particles. At this stage the van de Waals, hydrophobic and steric interactions dominate the system promoting aggregation. In the final state the repulsive interactions are greatly enhanced, likely with the exclusion of solvent molecules from the VLP structure. In other words, the electrostatic interactions are not screened in the final state. This suggests that during the process not only an increase of the size and molecular weight of the VLPs takes place, but also strong electrostatic changes over the surface of the particles are observed as time proceeds. It should be noted that B_22_ comprises a dichotomy of interactions, and none of them is excluded in the experiments performed, thus electrostatic repulsion is a significant but not the unique or sole contributor to the potential of mean force. Additionally, in order to support our results, it is of interest to examine in the future the consequences of changing the ionic strength in the measured hydrodynamic and electrostatic properties of the VLPs.

The results obtained from DLS, SLS and TEM experiments suggest that electrostatic interactions have much to do in the observed trends. The role of electrostatic interactions in the stability of viruses and VLPs has been explored in several experimental studies. From this point of view, the assembly in vitro and its relation with electrostatic interactions have been studied for a number of systems by changing pH, ionic strength and surfactant concentration [18,17,19–26]. From the experiments it is shown that the self-assembly process of many viral capsids is dominated by electrostatic protein-protein interactions and that significant change in surface charge can be induced by altering the pH and ionic strength of the solution. More interestingly, in some cases it has been probed that the EM of an empty capsid is very close to that containing RNA, a result that reflects that the surface electrostatic properties rely only on the capsomer and its distribution on the surface [17,25]. We have included in S4 Fig a calculation of the charge distribution of the L1 subunit using the Swiss-PdbViewer package for a better visualization of the electrostatic nature of the surface of the VLPs [45]. The top view of L1 in S4 Fig indicates a high concentration of negative charges in the surface of the VLP.

**Fig 6:**
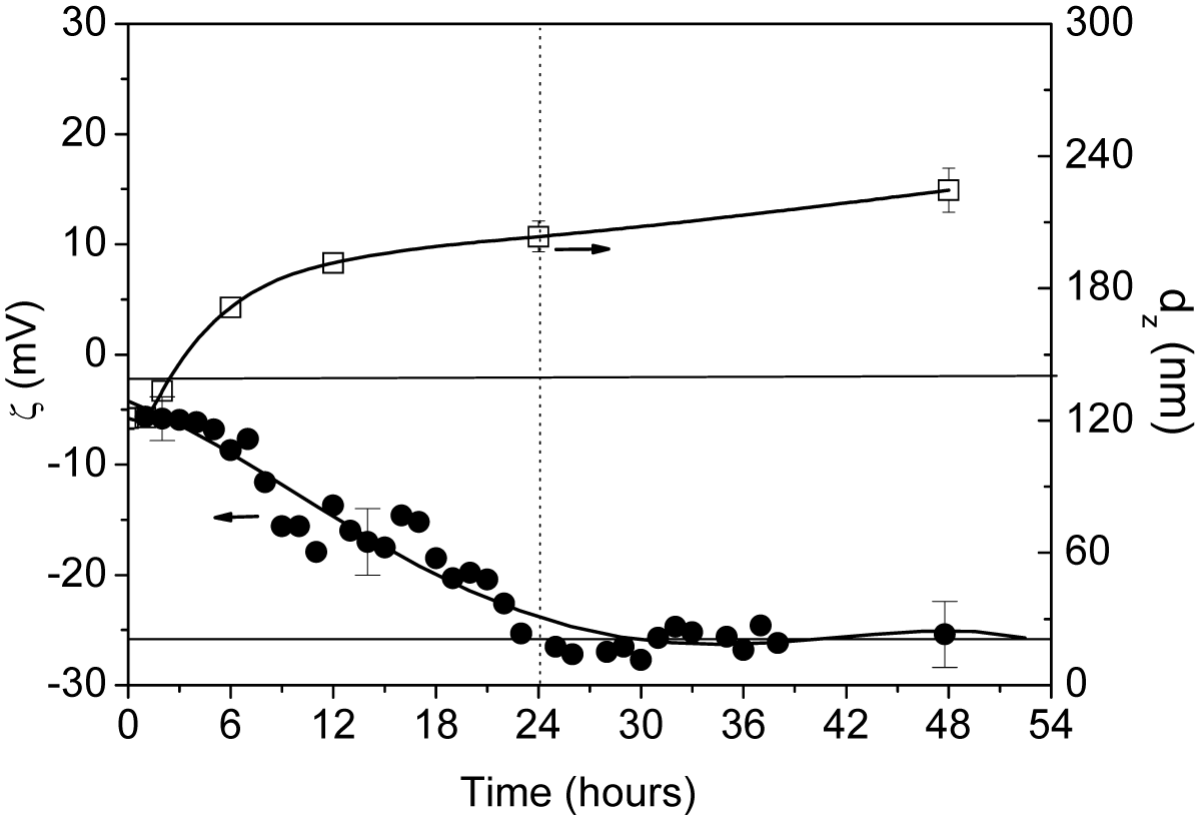
Results obtained from Electrophoretic Mobility experiments. Time evolution of (●) z-potential and (◻) z-average hydrodynamic radius at T = 298 K for the HPV16 VLPs studied. The error bars indicate the variability of experiments in batch mode performed at t = 0 h, 14 h (or 24h) and 48 h.

The results obtained from the EM measurements during the aggregation process are presented in Fig 6. In this case we have expressed the results as z-potential, ζ, obtained from the measured values of the electrophoretic mobility, μ_e_, using the Smoluchowski formula. This approach is likely applicable as the Debye length in 0.5 M NaCl is only 0.43 nm, which is 35 to 70-fold smaller than the particle radius, for initial and final samples, respectively [46]. As it can be observed, the values of ζ, measured by the unimodal analysis, sharply changes from an initial value of– 5.6 ± 1.5 mV to a final value of– 26.5 ± 3.0 mV. This later value compares very well with that obtained in the batch measurement,– 23.5 mV ± 1.0 mV, performed on a “fresh” VLP (48 h) sample (i.e. it has not suffered any previous measurement) using the general purpose analysis model. The standard deviation is obtained from three independent measurements of the initial (0 h) and final states (48 h). In S5 Fig some examples of the results obtained in EM experiments (phase plot) are given. The phase data show a closely flat signal in the initial VLP (0 h) sample due to the low value of the ζ, close to neutral. For the final VLP (48 h) samples an increase of the amplitude in the oscillation of the signal is clearly seen, in the experiments performed in both monomodal and general purpose modes. Additionally, a good reproducibility of the results is observed.

The trend in EM experiments goes parallel with that observed for the diffusion coefficient and the size of the VLPs. Thus an increase in the net charge during the stabilization process may be anticipated for the system under study. The trend is confirmed also from triplicate batch measurements (see the error bars in Fig 6) performed for the initial and final samples as well as for intermediate samples at time of 14 h for ζ and 24 h for D_z_. The results obtained suggest that the initial VLPs are slightly charged under the conditions of pH and ionic strength explored, and that the structural changes during the stabilization process cause a strong increase of the surface net charge due to the size increase and accommodation of additional L1 pentamers into the VLP in the final state. This increase observed in the net charge is in strong agreement with the change in the second virial coefficient observed previously, which in fact should be due to strong repulsive forces among the particles.

The question about the importance of electrostatic interaction has also been addressed from the theoretical point of view. Some recent theoretical models about the self-assembly of virus and VLPs take into account the balance between repulsive interactions (electrostatic), which acts against assembly, and binding interactions (van der Waals, hydrophobic or steric forces), which are the driving forces for the assembly. Šiber and Podgornik examined theoretically the balance between these interactions in the self-assembly process [16]. In this framework, the binding forces depend on the specific angle between protein subunits, or in other words on the specific structural 3D arrangement, a situation that gives rise to a predetermined self-assembly process. In highly salty solutions as those in the present study, the electrostatic interactions are screened, resulting in larger binding energy that favors aggregation (in agreement with a negative value of the second virial coefficient, B_22_, as occurs in our initial samples). An interesting result of Šiber and Podgornik model is that electrostatic interaction scales with the second power of the capsid radius, so a change in the energetic balance would be present during the process, as the size of the VLPs increases. This balance finally results in the formation of the viral capsids with a predetermined radius, given by the specific angles between the protein subunits, which ultimately give rise to specific electrostatic properties of the capsid surface in the final state. The process requires the reorganization of the subunits to be modulated by a fine balance between repulsive and attractive interactions. Taking into account this picture the changes observed in B_22_ and ζ indicate strong changes in the electrostatic features on the surface of VLPs, due to the aggregation and reorganization of the protein subunits. However, the theoretical approaches provide explanations about possible changes in electrostatic features that go beyond a simple counting of the number of charged residues on the surface of the particles [47,48]. It should be noted here that unlike rigid charged colloids, for which the relation between electrostatic properties and surface charge is straightforward, native virions and VLPs could be considered as “soft diffuse particles” [47]. In these systems electrostatic properties are highly dependent not only on the net charge on the surface, but also on charge distribution and permeability with respect to solvent and ions. This means that any change in the surface charge distribution might also lead to important changes in ζ for a given pH and ionic strength. The electrophoretic mobility is determined in this case by a delicate balance between the electrical force and electro-osmotic drag. In particular, the theoretical model developed by Duval et al. for diffuse soft particles shows that the screening of the electro-osmotic drag increases upon increasing the charge density and hydrodynamic permeability, thus resulting in a higher electrophoretic mobility [47]. In a different framework, by considering the case of hard spheres and the Debye-Hückel approximation, Lošdorfer-Božič and Podgornik [48] analyze how the symmetry of charge distribution modifies the interaction energy and find that local charge inhomogeneities affect differently the electrostatic interactions of overall equally charged particles.

Taking into account these theoretical approaches, the changes observed during the stabilization process of HPV16 VLP could be ascribed to both the increase of the hydrodynamic size of the VLPs and a reorganization of the capsomeres into the final mature structure. In the initial system, the electrostatic repulsion does not compensate the attractive interactions, which tend to aggregate the particles. This means that the VLPs are not stable and tend to increase in size. The size increase and likely the reorganization of capsid subunits into their preferred positions, would directly lead to a change of the surface properties. As judged by the results obtained, the system stabilizes when a determined size and surface charge (EM or ζ) is approached. The increase in size is then related to an increase of net charge over the surface. Such reorganization may be achieved either globularly within capsomeres or at local level for the L1 proteins. Also, other observations reported may be related to this reorganization, such as the open and closed VLP forms [49] and the flexible rearrangement of the extreme regions (last 20–30 residuals) of the lysine- and arginine-rich C-terminals [13]. The largely basic C-terminal disordered segment in L1 subunits is strongly involved in intercapsomeric interaction. A recent study reports that the C-terminal arm, loops out from the core of the L1 subunit, forms contacts (including a disulfide) with two subunits in a neighboring pentamer, and reinserts into the pentamer from which emanates the terminal. This segment is finally located in the innermost part of the VLP surface (see S6 Fig [50]), so we do not expect contributions of C-terminal arm to the electrostatic particularities of the outer surface of the final VLPs. It has to be noted that a major contribution to the strong repulsive force observed in the final state should come from the outer surface of the VLPs, and then from the exposed segments of the L1 pentamers. This outer surface is created by the protruding D-E, E-F and F-G loops (see S4 Fig). These loops, with an excess of electric charges, are extremely variable regions among different HPV types, and are known to interact with L2 protein [50,51]. These regions of the outer surface are the candidates for receptor interaction [50] and are capable of inducing conformational neutralizing antibodies [52–55].

## Conclusions

Based on our results obtained by dynamic and static light scattering, transmission electron microscopy and electrophoretic mobility, we conclude that strong electrostatic changes occur during the stabilization process of HPV16 virus-like particles. All these techniques provide us with different probes about the generation of stabilized virus-like particles after a time period of 48 hours at a temperature of 298 K. Dynamic light scattering mainly probes an important decrease of the diffusion coefficients during the process, which supports a 2-fold increase of the hydrodynamic size of the particles. Interestingly, the result obtained in static mode supports the presence of a clear colloidal instability of the initial virus-like particles, as revealed by a negative value of the second virial coefficient which is related to small repulsive electrostatic forces among the particles. This is in agreement with a small absolute value of the electrophoretic mobility and z-potential and, then, of the net surface charge. As time proceeds, and in parallel to the increase of the hydrodynamic size, strong enhancements of the z-potential and of the net surface charge (in absolute values) take place, suggesting enhanced repulsive electrostatic interactions and, consequently, a stabilized colloidal system. Transmission electron microscopy micrographs show that in the final state reached through the stabilization process the virus-like particles tend to show the characteristic 6-fold symmetry of the capsomeres, as expected in T = 7 icosahedral systems. The results obtained further show that electrophoretic mobility is a valuable methodology that can be applied to monitor the process, with interesting applications in vaccine developments, as it allows one to test readily the influence on VLP stability by both process and formulation impacts.

## Supporting Information

S1 Fig Cumulant analysis given by Eq (3) applied to DLS autocorrelation functions. (◻, black) 0 h, (○, red) 1 h, (△, green) 2 h, (▽, blue) 6 h, (◁, magenta) 12 h, (▷, cyan) 24 h and (◇, navy) 48 h. The parameters of the fits are given in Table 1.
(TIF)

S2 Fig DLS autocorrelation functions of the initial and final VLP samples. Red symbols correspond to VLP (0 h) and blue symbols to VLP (48 h). The measurements were performed with triplicate at T = 298 K.
(TIF)

S3 Fig DLS autocorrelation functions obtained for different aliquots of the samples. Open symbols corresponds to VLP (0 h) and closed symbols to VLP (48 h). The experiments were performed in the concentration range indicated. The solid lines correspond to the result obtained for the stock initial and final solutions. The measurements were performed in a low-volume quartz cuvette (Malvern Instruments ZEN2112) at T = 298 K.
(TIF)

S4 Fig Distribution of the electric charge at the molecular surface of a single subunit of L1 (PDB 1DZL) in the standard orientation. The molecular surface is colored with a red (negative cutoff– 0.8 kT/e), white (neutral points), blue (positive cutoff + 0.8 kT/e) color gradient in: A) side view and B) top view (exposed surface). The simplest calculation for the electrostatic potential (Coulomb) has been selected as a first approach, using the Swiss-PdbViewer package [45].
(TIF)

S5 Fig Phase plot obtained for different samples in EM measurements. The shorter experiments correspond to those obtained using the continuous monomodal analysis to follow the stabilization process for the initial VLP (red) and final VLP (green) samples. The longest experiment (blue) corresponds to a batch experiment performed in final “fresh” VLP samples (this sample has not been handled in any previous experiment). Sample concentration was 0.1 mg⋅mL^-1^ in 0.5 M NaCl with a pH value of 6. The sample was transferred to a zeta cell (Malvern Instruments DTS1060) and measured at T = 298 K using an applied voltage of 9 V.
(TIF)

S6 Fig Interpentamer L1 interaction, viewed (A) normal and (B) perpendicular to the five-fold axis (PDB 3J6R). The final 70 residues of the C-terminal in one of the L1 molecules (yellow) are indicated in cyan color (Swiss-Pdb Viewer package [45]). Note that the C-terminal of one L1 (yellow) in the pentamer interacts with the L1 (pink) outside this pentamer, while the C-terminal of the later L1 (pink) interacts with another L1 (blue). Consequently, the interactions contribute to the formation of L1 trimer.
(TIF)

S1 Table Values of z-, w- and n-average hydrodynamic diameters obtained from the cumulant analysis of the autocorretation functions obtained in DLS measurements. (DOCX)
