# Supplementary figures and images for: Evidences of Changes in Surface Electrostatic Charge Distribution during Stabilization of HPV16 Virus-Like Particles
Source: PLoS One. 2016 Feb 17;11(2):e0149009. doi: 10.1371/journal.pone.0149009 (PMC4757414; doi:10.1371/journal.pone.0149009)

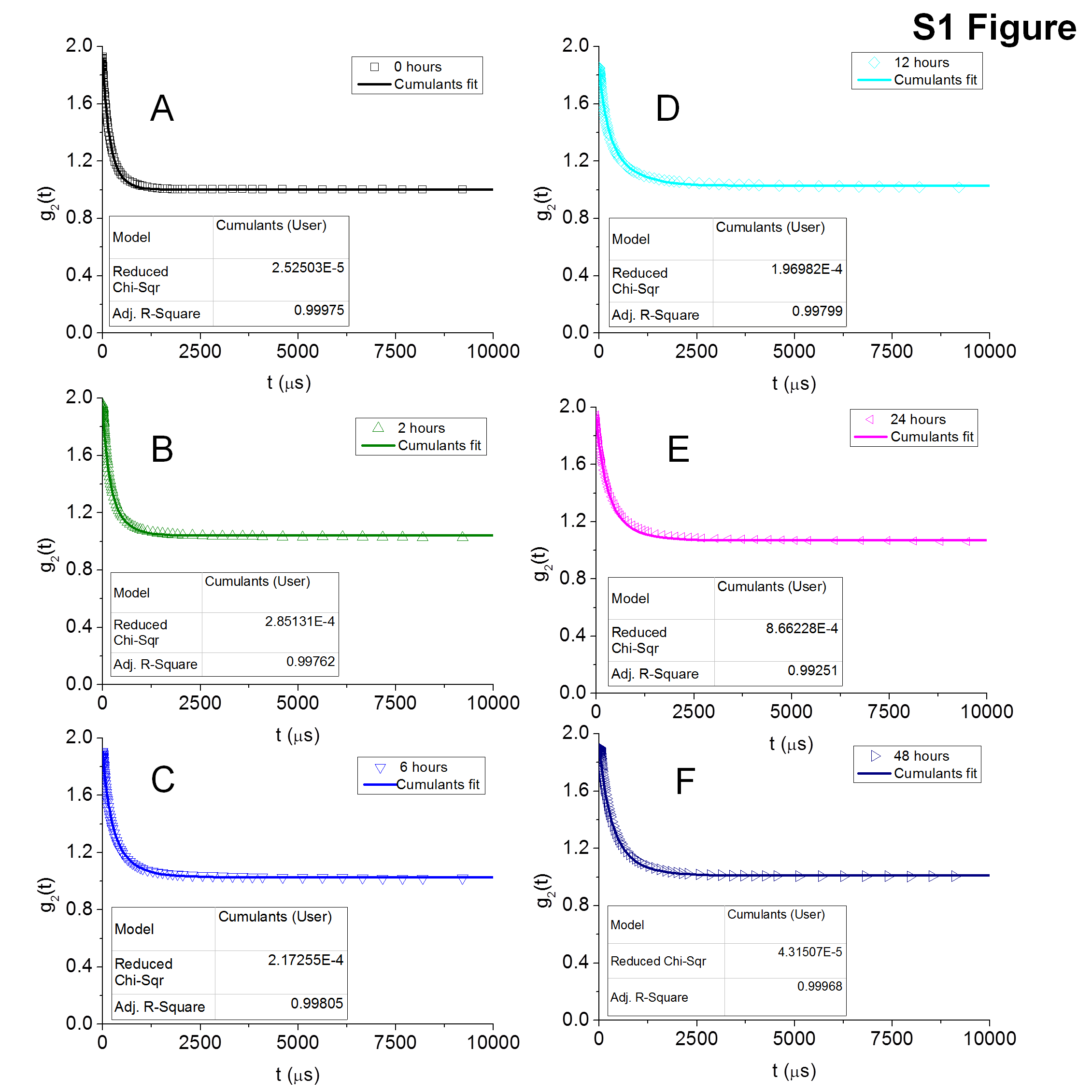

Supplement: S1 Fig — (◻, black) 0 h, (○, red) 1 h, (△, green) 2 h, (▽, blue) 6 h, (◁, magenta) 12 h, (▷, cyan) 24 h and (◇, navy) 48 h. The parameters of the fits are given in Table 1. (TIF) [file pone.0149009.s001.tif]

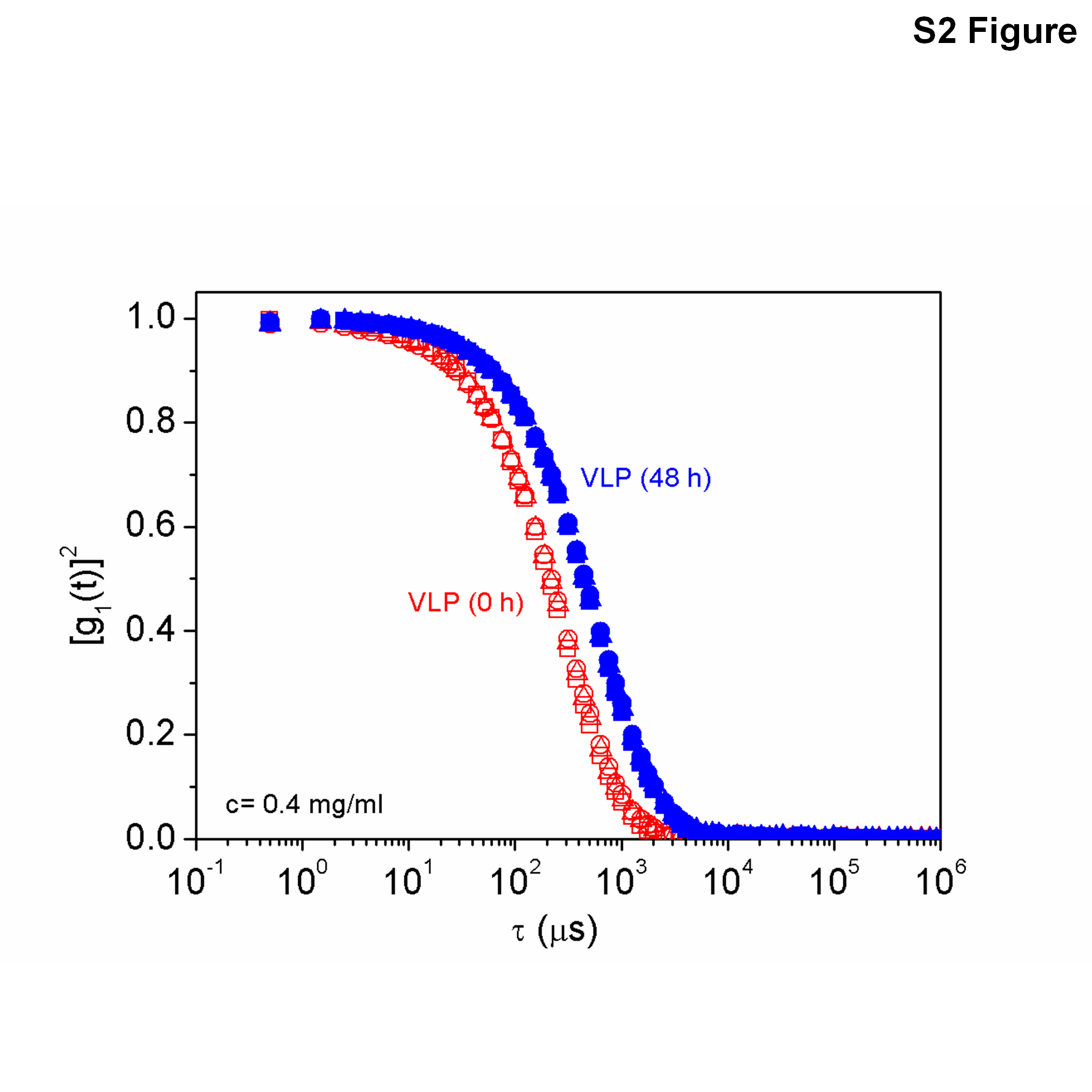

Supplement: S2 Fig — Red symbols correspond to VLP (0 h) and blue symbols to VLP (48 h). The measurements were performed with triplicate at T = 298 K. (TIF) [file pone.0149009.s002.tif]

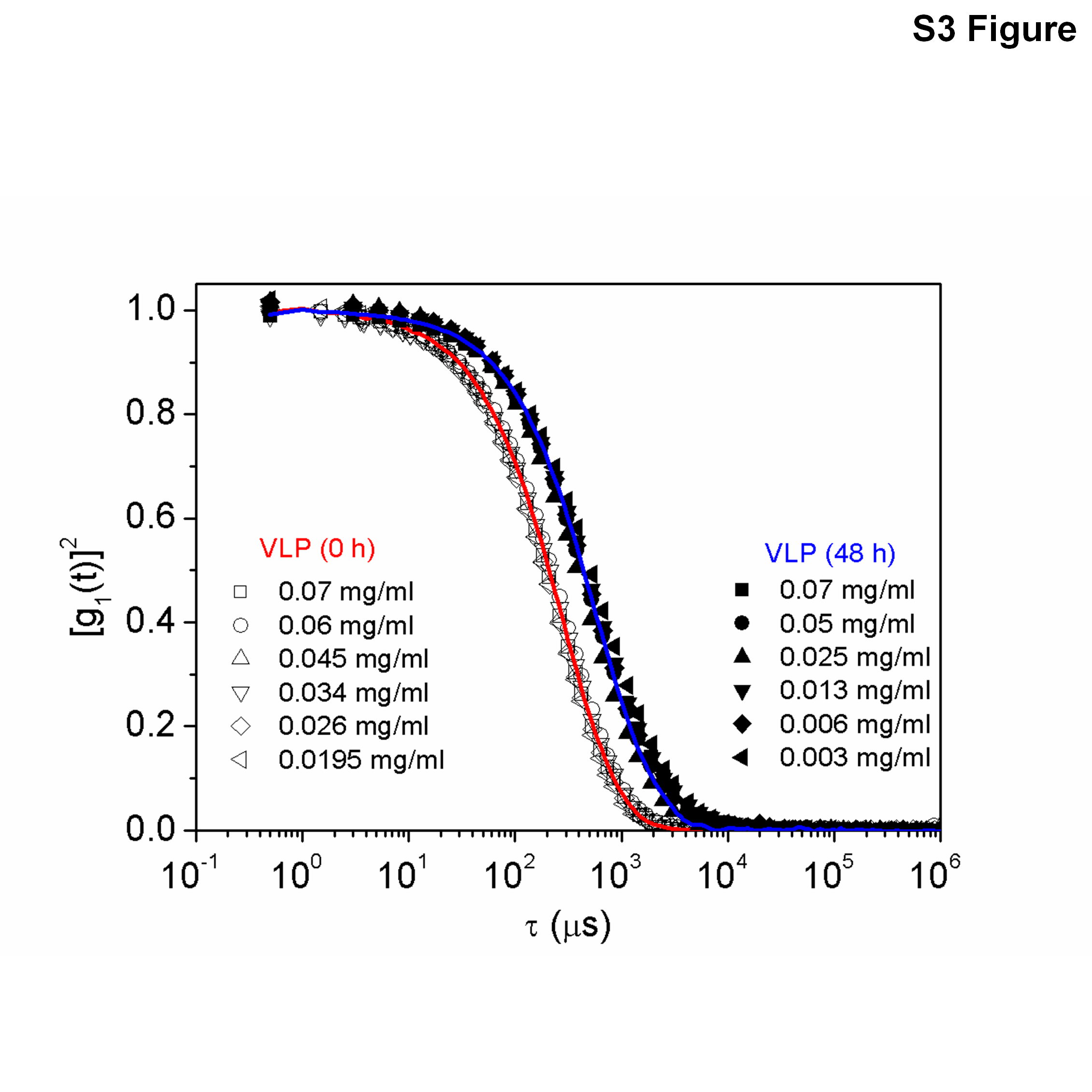

Supplement: S3 Fig — Open symbols corresponds to VLP (0 h) and closed symbols to VLP (48 h). The experiments were performed in the concentration range indicated. The solid lines correspond to the result obtained for the stock initial and final solutions. The measurements were performed in a low-volume quartz cuvette (Malvern Instruments ZEN2112) at T = 298 K. (TIF) [file pone.0149009.s003.tif]

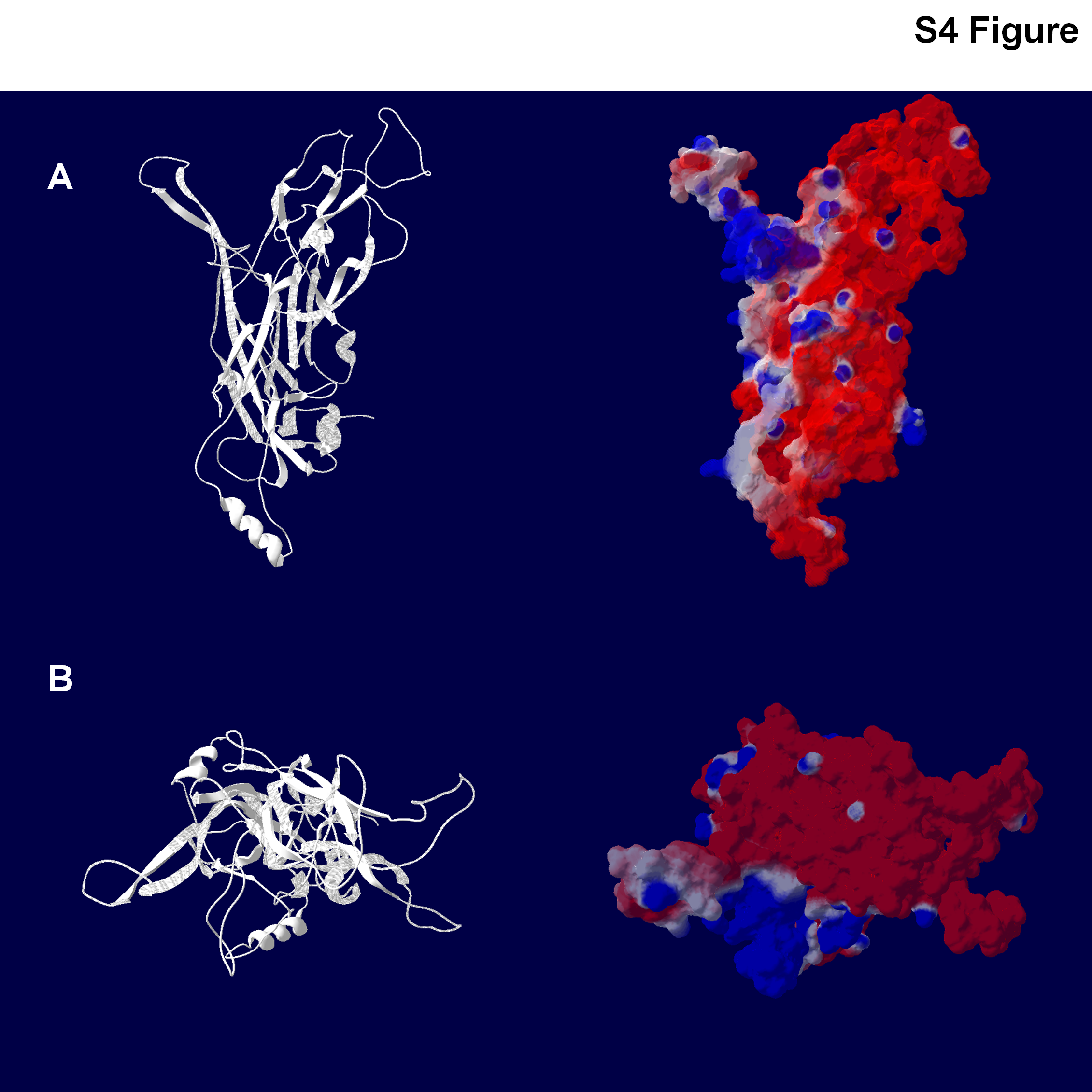

Supplement: S4 Fig — The molecular surface is colored with a red (negative cutoff– 0.8 kT/e), white (neutral points), blue (positive cutoff + 0.8 kT/e) color gradient in: A) side view and B) top view (exposed surface). The simplest calculation for the electrostatic potential (Coulomb) has been selected as a first approach, using the Swiss-PdbViewer package [45]. (TIF) [file pone.0149009.s004.tif]

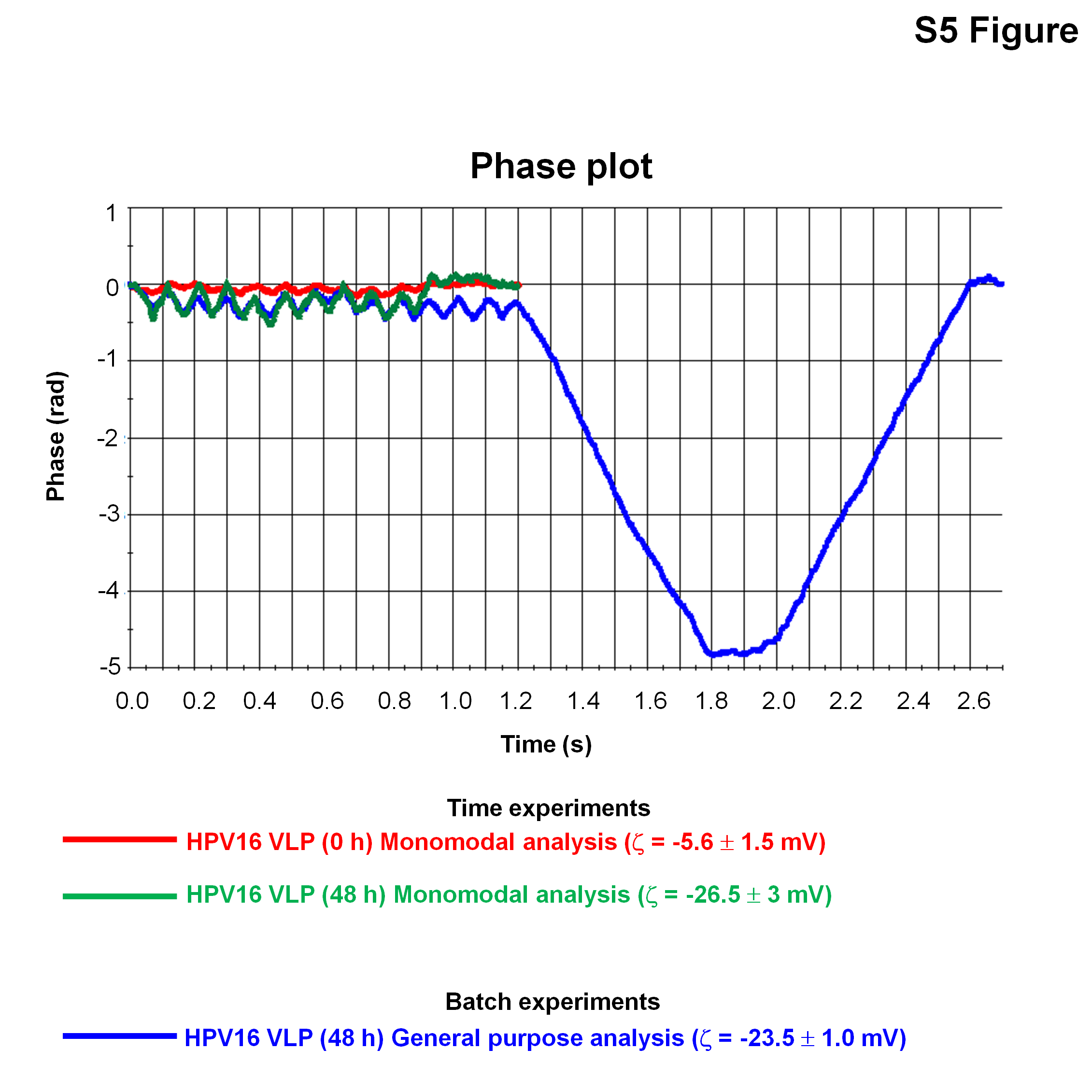

Supplement: S5 Fig — The shorter experiments correspond to those obtained using the continuous monomodal analysis to follow the stabilization process for the initial VLP (red) and final VLP (green) samples. The longest experiment (blue) corresponds to a batch experiment performed in final “fresh” VLP samples (this sample has not been handled in any previous experiment). Sample concentration was 0.1 mg⋅mL-1 in 0.5 M NaCl with a pH value of 6. The sample was transferred to a zeta cell (Malvern Instruments DTS1060) and measured at T = 298 K using an applied voltage of 9 V. (TIF) [file pone.0149009.s005.tif]

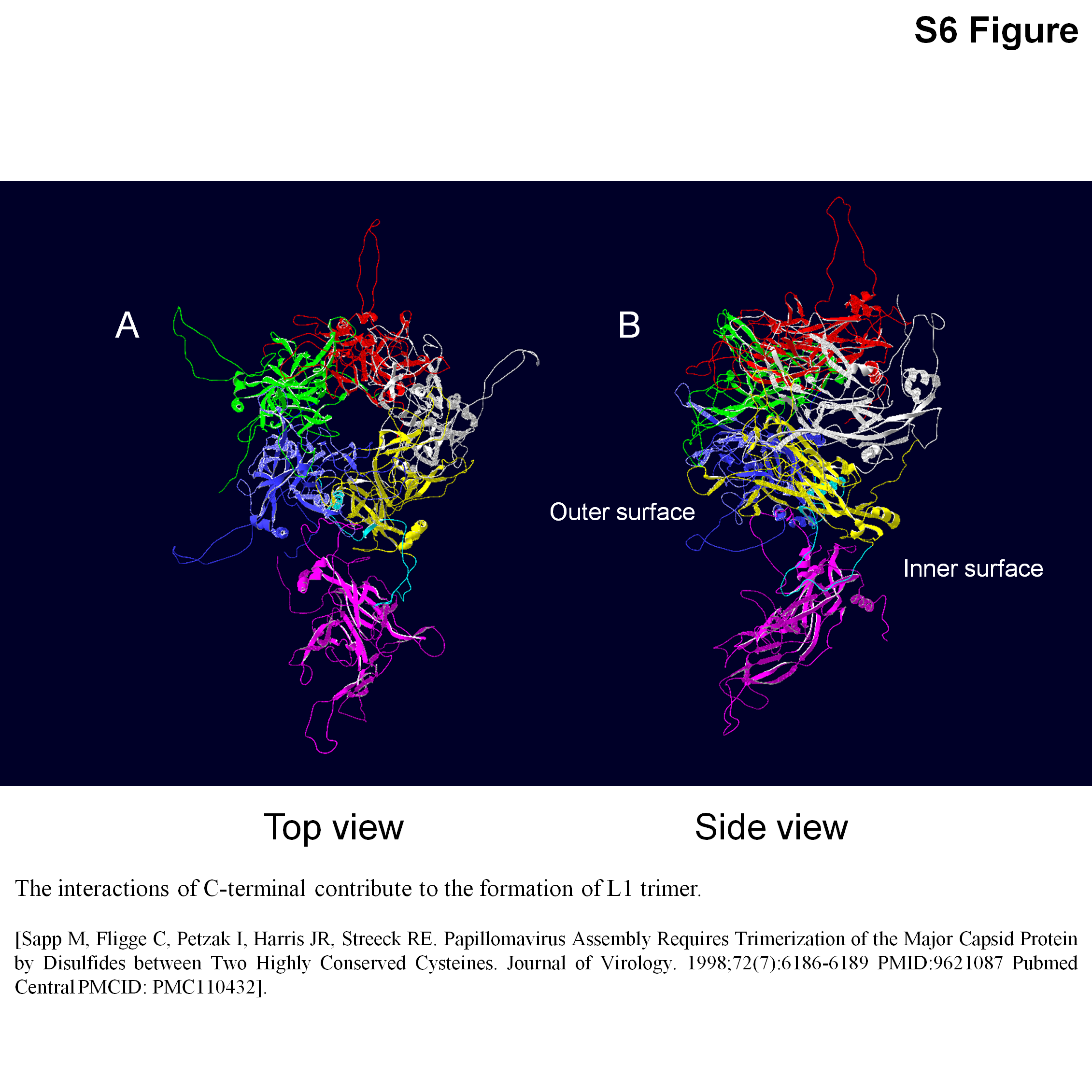

Supplement: S6 Fig — The final 70 residues of the C-terminal in one of the L1 molecules (yellow) are indicated in cyan color (Swiss-Pdb Viewer package [45]). Note that the C-terminal of one L1 (yellow) in the pentamer interacts with the L1 (pink) outside this pentamer, while the C-terminal of the later L1 (pink) interacts with another L1 (blue). Consequently, the interactions contribute to the formation of L1 trimer. (TIF) [file pone.0149009.s006.tif]
