# Supplementary material for: Evidences of Changes in Surface Electrostatic Charge Distribution during Stabilization of HPV16 Virus-Like Particles
Source: PLoS One. 2016 Feb 17;11(2):e0149009. doi: 10.1371/journal.pone.0149009 (PMC4757414; doi:10.1371/journal.pone.0149009)
Supplement: S1 Table — (DOCX) [file pone.0149009.s007.docx]

**S1 Table.** **Values of z-, w- and n-average hydrodynamic diameters obtained from the cumulant analysis of the autocorrelation functions obtained in DLS measurements.**

| **Sample** | **d_z_ (nm)** | **d_w_ (nm)** | **d_n_ (nm)** |
| --- | --- | --- | --- |
| 0 h | 117.9 | 74.6 | 37.5 |
| 1 h | 117.1 | 75.1 | 36.9 |
| 2 h | 133.4 | 75.4 | 34.1 |
| 6 h | 168.7 | 92.6 | 37.6 |
| 12 h | 196.1 | 107.6 | 43.7 |
| 24 h | 203.5 | 114.5 | 48.3 |
| 48 h | 226.1 | 131.9 | 58.8 |
